# Establishment of a Quadruplex RT-qPCR Method for the Detection of All Lineages of PPRV

**DOI:** 10.3390/ani16091397

**Published:** 2026-05-03

**Authors:** Jiao Xu, Jiani Li, Qinghua Wang, Jiamin Zhou, Shuang Liu, Yingli Wang, Jiarong Yu, Jingyue Bao, Lin Yang

**Affiliations:** 1 China Animal Health and Epidemiology Center, Qingdao 266000, China; xujiao@cahec.cn (J.X.); lijiani07300@163.com (J.L.); wangqinghua@cahec.cn (Q.W.); zjm2020925@163.com (J.Z.); liushuang@cahec.cn (S.L.); wangyingli@cahec.cn (Y.W.); yujiarong@cahec.cn (J.Y.); 2 College of Veterinary Medicine, Qingdao Agricultural University, Qingdao 266000, China; 3 College of Veterinary Medicine, Gansu Agricultural University, Lanzhou 730000, China

**Keywords:** peste des petits ruminants, quadruplex RT-qPCR, differential diagnosis, lineage

## Abstract

Peste des petits ruminants (PPR) is an infectious disease with high morbidity and mortality rates, and four distinct lineages have been discovered in different regions globally. In this study, a quadruplex RT-qPCR method capable of differentiating all four lineages of PPRV was established. By screening specific conserved regions across all viral genomes, we designed primers, as well as four TaqMan probes capable of distinguishing all lineages. The established method underwent validation of its relevant characteristics. The sensitivity of the detection method was determined by testing plasmid serial dilutions ranging from 10^8^ to 10^0^ copies/μL; results showed that the method could detect as few as 10 copies per microliter of PPRV. No cross-reactivity was observed among the four probes or with other common pathogens of goats and sheep. The coefficient of variation (CV) values for inter-assay and intra-assay repeatability of each probe were both below 2% (intra-assay: 0.11% to 0.98%; inter-assay: 0.18% to 1.95%), demonstrating excellent repeatability. Testing of 62 clinical samples also confirmed that the method could effectively detect and differentiate clinical samples of different PPR lineages. This method, for the first time, enabled the differentiation of all PPRV lineages in a single reaction, improving the detection efficiency of the PPR virus and providing robust technical support for the global PPR eradication program.

## Simple Summary

We developed a quadruplex RT-qPCR method which can detect all lineages of the peste des petits ruminants virus and validated its performance characteristics. This method enables the differentiation of all viral lineages in a single reaction, thereby improving detection efficiency. It shows no cross-reactivity with other common animal pathogens while maintaining excellent repeatability. Compared to existing methods, it yields consistent and highly accurate results for clinical samples. Therefore, this method can serve as a reliable differential diagnostic tool for peste des petits ruminants.

## 1. Introduction

Peste des petits ruminants (PPR) is a severe infectious disease caused by infection with a virus known as peste des petits ruminants virus (PPRV), primarily infecting small ruminants, with morbidity and mortality rates reaching as high as 100% [1]. As a transboundary infectious disease, PPR has been prevalent in Africa and Asia for decades, causing huge losses to local livestock industries. As a WOAH-listed notifiable disease, PPR has been targeted by FAO and WOAH for global control and eradication by 2030.

PPRV has only one serotype, which can be divided into four lineages [2]. Among them, lineage I circulates in Eastern Africa, although reports of this lineage have become scarce in recent years. Lineage II is also prevalent in Eastern Africa and is the dominant strain in several West African countries. Lineage III is primarily distributed in East Africa and the Arabian Peninsula. Lineage IV has the widest distribution. In recent years, in addition to circulating in Africa, it has also been the epidemic strain in most Asian countries that have reported PPR outbreaks [3]. Notably, in 2024, multiple European countries reported peste des petits ruminants outbreaks for the first time, and the epidemic continues to spread. Phylogenetic analysis has shown that the peste des petits ruminants virus circulating in some European countries also belongs to lineage IV [4]. China reported its first peste des petits ruminants outbreak in 2007; due to effective prevention and control measures, the outbreak was confined to a small area [5]. In 2013, the disease was re-introduced into China, and relevant studies have confirmed that the peste des petits ruminants virus strains introduced into China on both occasions also belong to lineage IV, although they fall into different sub-branches [6].

Early detection and accurate identification are crucial for the prevention and control of peste des petits ruminants. Since many countries and regions have implemented vaccination against peste des petits ruminants to establish immune barriers, ELISA detection of viral antibodies in these areas can only be used to evaluate immunization efficacy and cannot be relied upon for confirming peste des petits ruminants [7]. For the diagnosis of peste des petits ruminants, etiological diagnostic methods are widely used, and various detection technologies for peste des petits ruminants are currently emerging. In addition to traditional RT-qPCR, several novel rapid visual diagnostic methods have been developed in recent years, including RPA [8], MIRA [9], and CRISPR [10], among others. Compared with traditional techniques, these methods often feature shorter detection times, simple operation, and high visual clarity. However, most of them generally involve high costs, making them difficult to implement on a large scale in countries and regions with limited financial resources. Overall, RT-qPCR remains the most suitable technology for the diagnosis of PPR at present.

In addition to timely detection of the virus, the identification and typing of circulating strains is also an important component in the prevention and control of peste des petits ruminants. Once a non-endemic pathogen is introduced for the first time, it may cause high morbidity and mortality in the early stages of an outbreak due to the lack of population immunity [11]. Early detection and prompt interventions are crucial for minimizing losses. The traditional approach involves amplifying and sequencing specific viral fragments or the complete genome of detected PPRV, followed by phylogenetic analysis for typing [12]. This process often takes a considerable amount of time. However, the establishment of differential diagnostic methods can significantly shorten this process, and such differential diagnostic approaches have also emerged for PPR.

Relevant studies have shown that vaccination against peste des petits ruminants can result in transient viral shedding, although the shed virus is non-infectious [13]. Nevertheless, this can still interfere with the confirmation of peste des petits ruminants cases. Given that most of the attenuated vaccines currently used worldwide are derived from lineage II, Tang et al. established a detection method capable of distinguishing between lineage II and lineage IV, which is suitable for countries and regions where lineage IV is prevalent [14]. In response to the prevalence of lineage IV in Asia, we have also established an RT-qPCR detection method targeting this lineage, which exhibits good sensitivity and can detect up to six copies per microliter of virus [15]. However, although lineage IV is the predominant circulating strain globally, other lineages continue to persist in specific regions and pose a risk of spreading to other areas [16,17,18]. Therefore, developing an RT-qPCR detection method capable of simultaneously distinguishing all lineages would greatly enhance the applicability of such a differential diagnostic approach. Moreover, achieving typing of detected strains in a single reaction without the need for subsequent sequencing would significantly shorten the overall diagnostic process and greatly improve testing efficiency. Most of the existing detection methods for PPR are universal detection methods and often cannot distinguish between different lineages. Some detection methods can differentiate vaccine strains from wild-type strains, but no method has yet been able to distinguish all four viral lineages in a single reaction system. The development of this method fills this gap and is of great significance for epidemic surveillance; we hypothesized that, by targeting lineage-specific conserved regions in a single multiplex reaction, it is possible to develop a one-step quadruplex RT-qPCR assay that can not only detect but also distinguish all four lineages of peste des petits ruminants virus (PPRV) with high sensitivity and specificity. We further hypothesized that this novel assay would significantly outperform existing sequential or sequencing-based methods in terms of throughput and efficiency for PPRV surveillance in regions where multiple lineages co-circulate. The novelty of the present assay lies in two key aspects. First, it is the first RT-qPCR method capable of simultaneously detecting and distinguishing all four PPRV lineages (I, II, III, and IV) within a single reaction. Second, it achieves this without the need for post-amplification sequencing or sequential multiplex reactions, thereby significantly improving throughput and reducing turnaround time. This contrasts sharply with prior approaches, which required either separate reactions for each target or additional sequencing steps to achieve lineage identification. To test these hypotheses, by screening the conserved regions specific to each lineage, we designed four probes targeting different lineages. Through the optimization of reaction conditions, we established a reaction system comprising one pair of primers and four sets of probes, and validated the relevant characteristics of the method. This is the first RT-qPCR assay capable of distinguishing all four lineages in a single reaction.

## 2. Materials and Methods

### 2.1. Viruses and Plasmids

Plasmids of four lineages of PPRV used in this study were constructed and verified by Sangon Biotech (Shanghai) Co. Ltd. (Shanghai, China); each plasmid contains an inserted sequence of 449 bp, which starts from the forward primer (at nucleotide position 1735) and ends at the reverse primer (at nucleotide position 2183), and the concentration of each plasmid was determined using a NanoDrop spectrophotometer (Thermo Fisher Scientific, Waltham, MA, USA). Strains of PPRV lineage I to IV viruses, Goat pox virus (GPV), Orf virus (ORFV), Foot-and-mouth disease virus (FMDV) and lineage I to IV PPRV were stored at −80 °C in our laboratory.

### 2.2. Primer and Probe

A total of 93 genomic sequences representing all four PPRV lineages were collected from GenBank and aligned, information of sequences was shown in Table S1. The following criteria were applied to select conserved regions for primer design: (i) the region must be 100% conserved across all 93 sequences within the primer binding sites; (ii) the amplicon length should be between 300 and 500 bp to accommodate four lineage-specific probes; (iii) the region should flank a variable region containing lineage-specific single nucleotide polymorphisms (SNPs) to enable probe-based differentiation. For lineage-specific probe design, the following strategy was applied. First, conserved regions within each lineage were identified as candidate probe-binding sites. To ensure specificity, the probe-binding region for each lineage was required to differ from the corresponding region in non-target lineages by at least 2–3 nucleotides, particularly at the 5′ and 3′ ends of the probe. No intentional mismatches were introduced within the probe sequences themselves; instead, natural polymorphisms among lineages were exploited for specificity. In silico cross-reactivity was evaluated by aligning each probe against all 93 sequences using BLASTn to confirm no unintended matches to non-target lineages. Based on the above principles, one primer pair and four lineage-specific probes were designed for each lineage on the conserved region within the phosphoprotein (P) gene. Table 1 summarizes the primers and probes used in the present experiment and their positions; the amplicon size is 449 bp.

### 2.3. Establishment of the Reaction

Viral RNA was extracted using a viral DNA/RNA extraction kit (Hzymes Biotech, Shanghai, China). The amplification and detection were performed using Applied Biosystems QuantStudio 1 Plus Real-Time PCR System (Thermo Fisher Scientific, Waltham, MA, USA) using a one-step RT-qPCR kit (QV114-01, Vazyme, Nanjing, China). Each 25 μL reaction system comprised: 12.5 μL of animal detection U+ probe qPCR Super PreMix, 1 μL each of the forward and reverse primers (10 μM), 0.5 μL each of the four probes (10 μM), and 3 μL of viral RNA, 5.5 μL ddH_2_O. The reaction program settings are as follows: the reverse transcription step was carried out at 50 °C for 10 min, after which the reverse transcriptase was inactivated and the DNA polymerase was activated at 95 °C for 5 min. This was followed by 40 amplification cycles, each consisting of denaturation at 95 °C for 15 s and annealing at 60 °C for 30 s. Fluorescence signals were collected from four channels, corresponding to the respective probes. All reactions were performed in triplicate (n = 3) for each sample within a single run, and the entire experiment was repeated three independent times (three runs) on different days to assess reproducibility.

### 2.4. Data Analysis and Interpretation Criteria

A sample was considered positive if the amplification curve showed a typical S-shape and the Ct value was ≤39.15 (Cut-off value). A sample was considered negative when no amplification curve was detected or the Ct value was >39.15. For lineage assignment, the four lineage-specific probes (labeled with FAM, VIC, CY5 and ROX) were analyzed in the same well. A sample is considered positive for a given lineage when the lineage-specific fluorescence signal is detected, and the Ct value and amplification curve satisfy the interpretation criteria. Regarding the handling of amplification curves, the baseline was set automatically by the real-time PCR software and, when necessary, manually adjusted to exclude background fluorescence from the early cycles. The threshold line was placed in the middle of the exponential phase of the amplification curves, typically at a ΔRn value as determined automatically by the software. All raw data were reviewed manually to confirm that each positive curve showed a clear exponential increase and a plateau phase; curves with unspecific background, a shallow slope or no plateau should be discarded and the sample was re-tested.

### 2.5. Specificity Test

We used the established method to detect PPRV lineages I to IV, as well as common sheep or goat viruses such as ORFV, GPV, and FMDV, in order to evaluate the specificity of the method.

### 2.6. Sensitivity and Repeatability Tests

To assess the analytical sensitivity of the assay, we used pre-constructed plasmids containing PPRV sequences. Plasmids were serially diluted tenfold from 10^8^ to 10^0^ copies·μL^−1^. Ct values amplified by different probes at various concentrations, along with those from negative controls, were collected to assess method sensitivity and generate a standard curve. For repeatability testing, we selected plasmids of PPRV lineages I–IV at high (10^7^ copies·μL^−1^), medium (10^5^ copies·μL^−1^), and low (10^3^ copies·μL^−1^) concentrations to evaluate repeatability.

### 2.7. Field Sample Detection

We validated the established detection method using 62 clinical samples collected previously; the nucleic acids for lineage I and lineage III were derived from viral isolates obtained from clinical samples, which were provided by the WOAH Reference Laboratory for Peste des Petits Ruminants. These samples had all been confirmed as PPRV nucleic acid positive or negative via RT-qPCR [19], and the lineages of the positive samples had been determined by sequencing. The reliability of our newly established method was then assessed based on its detection results.

## 3. Results

### 3.1. Multiple Sequence Alignments of the Primers and Probe

**Figure 1:**
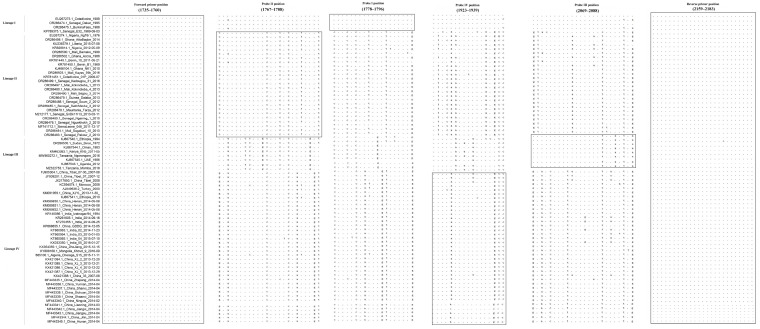
Alignment of the primer and probe targeted to the gene among multiple PPRV strains. The sequences of the forward primers, probe, and reverse primer are shown in boxes. The designed primers mapped well with most epidemic PPRV strains; the probe-binding regions for different lineages are highly conserved among the corresponding lineage strains.

The designed primers and probe-binding regions for different lineages are highly conserved among the corresponding lineage strains, which demonstrated that both the primers and probes developed in this research possess the necessary characteristics for use as a differential diagnostic method (Figure 1).

### 3.2. Specificity of the Developed Method

**Figure 2:**
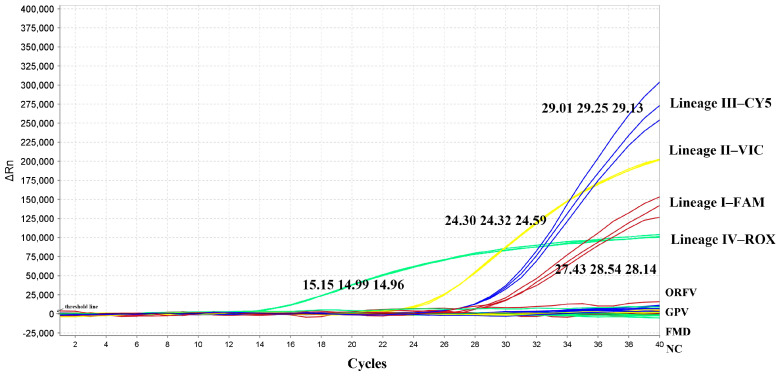
Specificity test of the RT-qPCR assay. All probes can only detect their corresponding peste des petits ruminants virus lineages without any cross-reactivity with each other or with other viruses; the Ct values for the detection of different lineages have been marked on the figure.

Each probe detected only its corresponding lineage with no cross-reactivity among probes. No amplification was observed for ORFV, GPV, FMDV, or the negative control (Figure 2 and Table 2).

### 3.3. Sensitivity of the Developed Method

**Figure 3:**
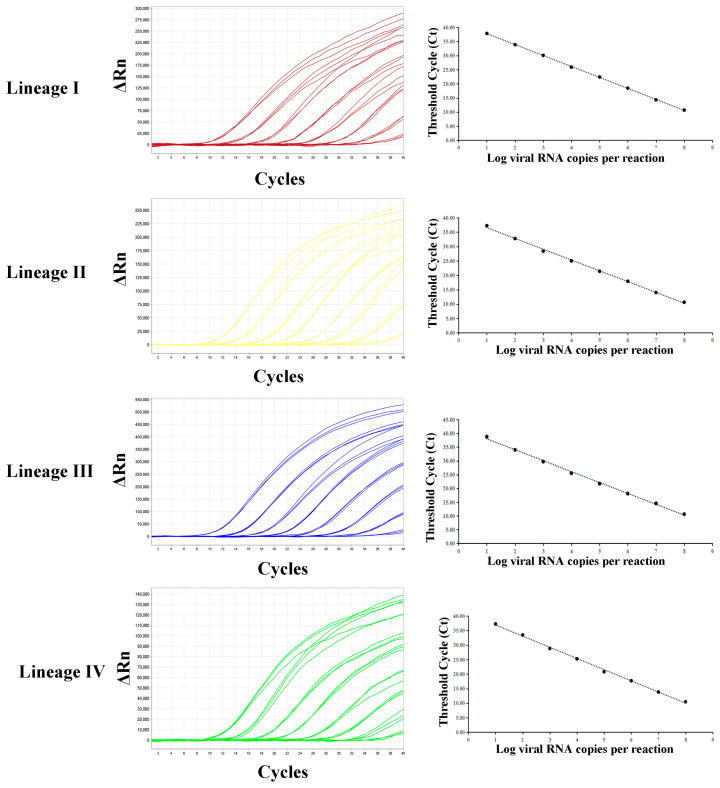
Sensitivity tests and standard curve for quadruplex RT-qPCR. All probes were capable of detecting as few as 10^1^ copies·μL^−1^. The standard curve for the quadruplex RT-qPCR assay was constructed; the correlation coefficients for lineage I to IV were all higher than 0.99.

All probes were capable of detecting as few as 10^1^ copies·μL^−1^. No Ct values were obtained at 10^0^ copies·μL^−1^ or for ddH_2_O. The standard curve of quadruplex RT-qPCR assay was constructed; the correlation coefficients for lineage I to IV were all higher than 0.99 (I: 0.999, II: 0.998, III: 0.998, IV:0.998) (Figure 3). The efficiencies for lineages I to IV were 95.8%, 95.2%, 96.5%, and 93.6%, respectively (Table 3).

### 3.4. Repeatability of the Developed Method

As summarized in Table 4, different lineages of PPRV were detected for the repeatability test under various concentrations. All tested lineages showed coefficients of variation (CV) below 2% (inter-assay) and 1% (intra-assay).

### 3.5. Detection of Field Samples

**Figure 4:**
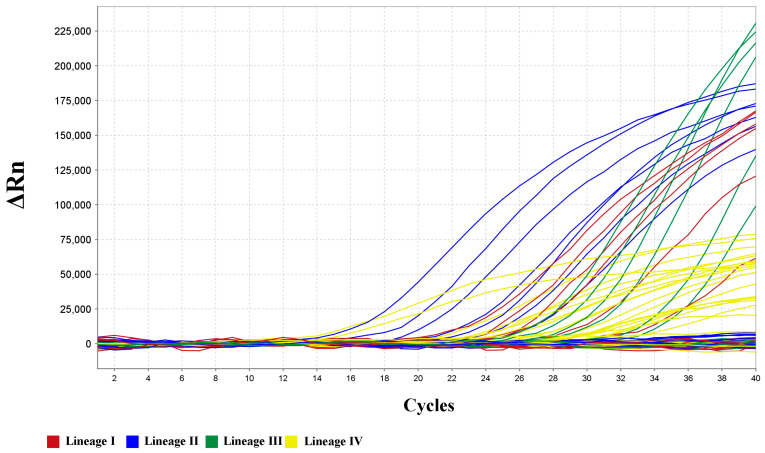
Detection of field samples. All positive samples were successfully detected and PPRVs of different lineages were also fully differentiated.

A total of 62 clinical samples, including 24 PPRV-negative and 38 positive samples covering all lineages, were collected in recent years. The detection results are shown in Figure 4 and Table 5; all 38 positive samples were successfully detected, and the PPRVs of different lineages were also fully differentiated (Table 6). The Ct value range for lineage I is 21.29 to 32.33, lineage II is 16.32 to 25.00, lineage III is 22.70 to 31.30, and lineage IV is 9.72 to 31.19. It is 100% consistent with the known background information.

### 3.6. Comparison of the Consistency Between the Results of This Assay and the Reference Method

**Figure 5:**
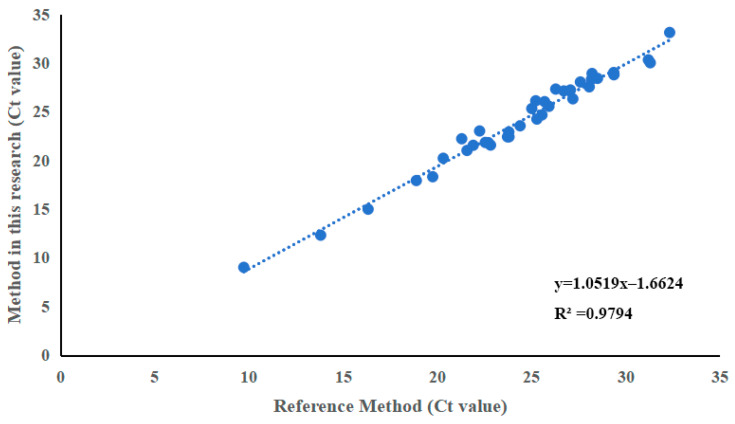
Comparison of the consistency between the results of this assay and the reference method. The Ct values obtained by the new assay showed excellent agreement with those from the reference RT-qPCR, with R^2^ > 0.9.

A total of 62 clinical samples were tested by both the newly developed assay and the reference RT-qPCR method. All samples that tested positive by the reference method were detected by this newly established method, yielding a relative detection rate of 100% for this method; a strong positive correlation was observed between the Ct values obtained by the two methods (Figure 5) (R^2^ = 0.979). These results demonstrate that the newly developed assay is comparable to the reference method for Ct value quantification.

### 3.7. Evaluation of Diagnostic Accuracy

To assess the clinical diagnostic accuracy of the newly developed quadruplex RT-qPCR assay, results were compared against Sanger sequencing as the gold standard for all 62 samples. As summarized in Table 7, the assay demonstrated a diagnostic sensitivity of 100% (95% CI: 90.7–100%) and a diagnostic specificity of 100% (95% CI: 94.2–100%). The overall percent agreement was 100% (62/62). The Cohen’s kappa coefficient was 1.00 (95% CI: 0.93–1.00), indicating perfect agreement between the new assay and the gold standard.

## 4. Discussion

As one of the most destructive transboundary animal diseases (TADs), PPR places a severe socioeconomic burden on developing regions in particular [20]. In response, FAO and WOAH launched the Global Eradication Program (GEP) in 2015, building on the earlier PPR Global Control and Eradication Strategy, with the goal of eradicating the disease worldwide by 2030 [21]. The success of the GEP hinges on accurate, timely, and accessible diagnostic tools. Laboratory testing plays a foundational role in early detection, outbreak response, and surveillance, which are key components of coordinated control and eradication efforts. Within the PPR Monitoring and Assessment Tool (PMAT), diagnostics serve as a critical indicator for assessing national capacities and monitoring progress along the eradication pathway [22]. Reliable diagnostic systems are vital for confirming clinical cases, certifying freedom from disease, and validating surveillance data, thereby supplying the evidence necessary to transition from control phases to eradication.

Laboratory testing for PPR typically includes virus isolation, etiological detection, and serological detection. Among these, virus isolation is considered the gold standard for diagnosing PPR, as the infection can be determined by observing cytopathic effects (CPE) on Vero or Vero-SLAM cells (expressing the SLAM/CD150 receptor) after inoculating with pathological samples [23]. However, this diagnostic method requires high technical proficiency and is time-consuming, making it unsuitable for large-scale testing. The detection of antibodies is critical for determining prior exposure to PPRV, tracking vaccine-induced immunity, and gauging protection at the population level. Such methods offer valuable information on historical infection, vaccination status, and overall herd immunity. They therefore serve a vital function in sero-surveillance and efforts to eradicate PPR [21]. ELISA is the most commonly used technique for serological testing, primarily including blocking ELISA (bELISA) and competitive ELISA (cELISA). These assays primarily target antibodies against viral nucleoprotein (N) or haemagglutinin (H). Due to their high sensitivity and specificity, they are widely employed for serological diagnosis, surveillance, and monitoring. Because they cannot differentiate vaccinated animals from naturally infected ones, these methods are generally not applicable for diagnosing PPR infection in animals; they remain indispensable for routine monitoring and post-vaccination evaluation [24]. However, for animals that are confirmed to have never been vaccinated, the presence of viral antibodies can be interpreted as indicating current infection or past exposure to PPRV.

Nucleic acid-based assays detect viral RNA with high sensitivity and specificity. By targeting conserved regions of PPRV genes (N, F, H, or P), they enable early diagnosis of even mild or subclinical infections while distinguishing PPRV from related morbilliviruses. Compared with RT-PCR, RT-qPCR is a key tool for confirming PPR outbreaks, offering more rapid, sensitive, and specific detection of viral RNA. It can complete the entire testing process within a few hours, with a detection limit as low as a few copies. More importantly, its low cost makes it highly suitable for large-scale laboratory testing. This capability facilitates timely interventions and helps curb the spread of PPRV, as evidenced by many outbreaks in different countries. Currently, a range of RT-qPCR methods targeting viral genes including N, F, and P have been developed, demonstrating the limits of detection (LODs) of 10–32 genomic copies [25,26,27,28]. This has also given rise to multiplex RT-qPCR assays that can detect multiple pathogens at once. Settypalli et al. established a multiplex RT-qPCR assay which enables the simultaneous detection of multiple pathogens, such as PPRV, Capripoxvirus, Pasteurella multocida, and Mycoplasma capricolum ssp. capripneumoniae, providing a cost-effective and time-saving tool for differential diagnosis [29]. However, a multiplex RT-qPCR method for distinguishing between different lineages of PPRV has not yet been developed.

This study established a quadruplex RT-qPCR assay capable of simultaneously distinguishing four lineages of PPRV. Compared with common duplex and triplex detection methods, this approach offers higher throughput; moreover, when the sample volume is limited (such as clinical swabs, tissue homogenates, or environmental samples), this quadruplex PCR requires only one sample for multiple detections, thereby avoiding issues related to insufficient sample volume or subsampling errors. Our method utilizes only one pair of primers, which significantly reduces the likelihood of dimer formation and hairpin structures commonly associated with multiple primer pairs in traditional multiplex PCR, thereby enhancing the stability of the method [30].

Our validation results indicate that the detection sensitivity of this method can reach 10 copies per microliter, which is slightly lower compared to singleplex RT-qPCR that can detect single-digit copies. This is because accommodating four TaqMan probes necessitated a moderate increase in amplicon size, which slightly reduces the sensitivity [31,32]. Nevertheless, it remains sufficient for routine testing applications. The specificity results also demonstrated that the four probes could only recognize their corresponding lineages of peste des petits ruminants virus, with no cross-reactivity among them or with other viruses. This is crucial for ensuring the accuracy of the detection results. Detection results of clinical samples also demonstrated that this method can identify and detect all lineages of PPRV. To our knowledge, this is the first study to develop a single-reaction quadruplex RT-qPCR assay capable of simultaneously detecting and unequivocally differentiating all four lineages of peste des petits ruminants virus (PPRV). A comparison of our assay with previously reported PPRV detection methods is summarized in Table 8.

This method holds significant value for the surveillance of PPR in regions with different epidemic situations. In areas where multiple lineages are circulating (especially in African countries), it enables the rapid identification of the lineage of the virus from positive samples without the need for subsequent sequencing. In regions where the circulating lineage is relatively single, this method can be used as a routine PPR surveillance tool, allowing for the immediate detection of any introduction of other lineages and facilitating the prompt implementation of effective measures. In conclusion, as a detection method that is challenging to design, the establishment of this quadruplex RT-qPCR assay holds great significance for the global eradication of PPR.

Despite the promising performance of the newly developed assay, several limitations should be acknowledged. First, the sensitivity of the assay was determined using plasmid standards rather than in vitro transcribed RNA or live virus. Plasmid DNA is generally more stable and amplifies more efficiently than RNA, which may lead to an overestimation of the analytical sensitivity when applied to clinical RNA samples. Second, the specificity panel included a relatively limited number of pathogens (GPV, FMDV, ORFV). Although no cross-reactivity was observed, the assay has not been tested against a broader range of viruses that may be present in field samples, such as other paramyxoviruses or ruminant pathogens. Third, the number of clinical samples positive for lineage I and lineage III was limited due to their restricted geographic distribution and low prevalence. Further validation using a larger and more diverse set of field samples, particularly for these rare lineages, is warranted. Besides this, another potential limitation of this assay is the risk of mutations occurring in the probe-binding regions of circulating field strains. As an RNA virus, PPRV is prone to genetic evolution, and nucleotide substitutions, insertions, or deletions could arise in the regions targeted by our lineage-specific probes. Such mutations may reduce or abolish probe-binding, leading to false-negative results or incorrect lineage assignment. Although the primer and probe-binding regions were selected based on conserved sequences across 93 publicly available genomes representing all four lineages, and no mismatches were observed in our field samples, the continuous surveillance of emerging PPRV strains is warranted. Regular monitoring of the target regions and periodic updates of the probe sequences may be necessary to maintain the long-term accuracy and reliability of this assay, especially as eradication efforts progress and viral populations may change.

## 5. Conclusions

All experimental results indicate that our established method can effectively detect four different lineages of peste des petits ruminants virus in a single reaction. The method is stable, highly sensitive, and specific, greatly improving the efficiency of differential diagnosis of peste des petits ruminants virus, further validation using larger and more geographically diverse clinical sample panels would help to strengthen the field applicability of this assay.

## Figures and Tables

**Table 1:** Primers, probes and their positions used in this study.

	Sequence (5′-3′)	Target Lineage	Target Coordinates	Fluorophore	Quencher	Final Concentration (μM)	Probe Channel	Amplicon Size (bp)
Forward primer	TTATAAAAAACTTAGGACCCAGGTCC	/	1735–1760	/		0.4	/	449
Reverse primer	GCATCCTCGAGTCCTCTAATCTCTT		2159–2183			0.4		
Probe I	AACTCTAGCCAACCGAGCA	Lineage I	1778–1796	FAM	BHQ1	0.2	FAM	/
Probe II	CCRCACATCGACACCCCAGTCAAT	Lineage II	1767–1788	VIC	BHQ1	0.2	VIC	
Probe III	ACGATGACAGTGAGGCTGGACTCA	Lineage III	2069–2088	CY5	BHQ2	0.2	CY5	
Probe IV	GCTTAGCCCCTCGGGCC	Lineage IV	1923–1939	ROX	BHQ2	0.2	ROX	

**Table 2:** Specificity of developed method.

No.	Name of Strains	GenBank Accession Number	Lineage	Ct Value (FAM)			Ct Value (VIC)			Ct Value (CY5)			Ct Value (ROX)			Viral Load Copies·μL^−1^
1	China/XJYL/2013	KM091959	IV	39.35	/	39.27	/	/	/	/	/	/	15.15	14.99	14.96	1.00 × 10^7^
2	PPRV/Sudan/Sinar/1972	OR286505	III	/	/	/	/	/	/	29.01	29.25	29.13	/	/	/	3.40 × 10^3^
3	Nigeria 75/1	HQ197753	II	/	/	/	24.30	24.32	24.57	/	/	/	39.58	/	/	2.40 × 10^4^
4	PPRV/Cote_d Ivoire/1989	OL741724	I	27.43	28.54	28.14	/	/	/	39.48	39.56	/	/	/	/	2.50 × 10^3^
5	GPV	/	/	/	/	/	/	/	/	/	/	/	/	/	/	3.25 × 10^4^
6	ORFV	/	/	/	/	/	/	/	/	/	/	/	/	/	/	6.50 × 10^5^
7	FMDV	/	/	/	/	/	/	/	/	/	/	/	/	/	/	8.55 × 10^5^
8	H_2_O	/	/	/	/	/	/	/	/	/	/	/	/	/	/	/

**Table 3:** The efficiencies of established method.

Lineage	Standard Curve Equation	Slope	R^2^	PCR Efficiency (%)
Lineage I	y = −3.43x + 40.01	−3.43	0.999	95.8%
Lineage II	y = −3.44x + 40.31	−3.44	0.998	95.2%
Lineage III	y = −3.41x + 41.28	−3.41	0.998	96.5%
Lineage IV	y = −3.48x + 40.37	−3.48	0.998	93.6%

**Table 4:** Repeatability test results (n = 3).

	Lineage I			Lineage II						Lineage III			Lineage IV		
Plasmid Copy Number Copies·μL^−1^	Intra-Assay Precision			Intra-Assay Precision			Intra-Assay Precision			Intra-Assay Precision			Intra-Assay Precision		
	Mean	SD	CV (%)	Mean	SD	CV (%)	Mean	SD	CV (%)	Mean	SD	CV (%)	Mean	SD	CV (%)
1 × 10^7^	14.96	0.12	0.782%	15.62	0.04	0.246%	15.74	0.14	0.915%	15.47	0.15	0.984%	15.74	0.14	0.915%
1 × 10^5^	22.28	0.18	0.788%	23.40	0.22	0.945%	22.27	0.03	0.117%	23.45	0.05	0.192%	22.27	0.03	0.117%
1 × 10^3^	30.95	0.04	0.128%	30.22	0.09	0.297%	30.41	0.05	0.163%	31.53	0.08	0.265%	30.41	0.05	0.163%
				Lineage I			Lineage II			Lineage III			Lineage IV		
Plasmid copy number copies·μL^−1^				Inter-assay Precision			Inter-assay Precision			Inter-assay Precision			Inter-assay Precision		
				Mean	SD	CV (%)	Mean	SD	CV (%)	Mean	SD	CV (%)	Mean	SD	CV (%)
1 × 10^7^				15.20	0.21	1.36%	14.75	0.29	1.95%	16.21	0.06	0.40%	15.47	0.04	0.26%
1 × 10^5^				23.38	0.36	1.54%	21.88	0.19	0.88	23.13	0.15	0.64%	22.36	0.04	0.18%
1 × 10^3^				31.88	0.14	0.44%	29.23	0.19	0.66%	31.11	0.21	0.68%	30.26	0.11	0.37%

SD, standard deviation; CV, coefficient of variation.

**Table 5:** Information of clinical samples and detective results of RT-qPCR.

Sample ID			Type of Samples			RT-qPCR (Ct Value)			Lineage			Collection Date and Geographic Origin			Animal Species		
1	2	3	lymph node	swab	lung	21.90	23.72	25.92	IV	II	IV	2007.8 Tibet	2022.3 Xinjiang	2018.5 Jiangsu	Ga *	G *	S *
4	5	6	WOAH *	swab	WOAH	26.28	27.06	25.28	III	IV	I	/	2020.5 Liaoning	/	/	S	/
7	8	9	bronchus	lung	spleen	-	22.82	29.37	-	IV	IV	2015.4 Guizhou	2017.3 Hunan	2017.3 Hunan	S	G	G
10	11	12	lymph node	WOAH	spleen	23.80	21.29	27.59	IV	I	IV	2018.2 Qinghai	/	2016.11 Ningxia	G	/	S
13	14	15	spleen	lymph node	spleen	-	-	-	-	-	-	2014.6 Shaanxi	2014.6 Shaanxi	2014.5 Yunnan	S	S	G
16	17	18	bronchus	WOAH	WOAH	21.57	24.39	13.80	IV	III	IV	2014.4 Heilongjiang	/	2014.4 Heilongjiang	G	S	G
19	20	21	swab	lymph node	WOAH	28.18	-	-	IV	-	-	2014.4 Sichuan	2014.4 Sichuan	2014.4 Sichuan	S	S	S
22	23	24	lymph node	swab	WOAH	31.20	25.22	29.37	IV	IV	III	2014.4 Chongqing	2014.4 Chongqing	/	G	S	G
25	26	27	bronchus	bronchus	lung	16.32	-	28.21	II	-	IV	2025.2 Xinjiang	2025.2 Xinjiang	2025.2 Xinjiang	G	S	S
28	29	30	swab	WOAH	swab	22.24	26.71	27.19	IV	II	IV	2025.2 Xinjiang	2025.2 Xinjiang	2015.4 Guizhou	S	S	G
31	32	33	lymph node	spleen	spleen	9.72	28.06	-	IV	IV	-	2014.2 Ningxia	2014.2 Ningxia	2014.2 Ningxia	S	G	G
34	35	36	WOAH	lung	bronchus	-	19.75	-	-	IV	-	2014.4 Hubei	2014.4 Hubei	2018.5 Jiangsu	S	S	S
37	38	39	lymph node	lymph node	WOAH	29.36		28.50	IV	-	I	2018.5 Jiangsu	2018.5 Jiangsu	/	G	S	/
40	41	42	WOAH	WOAH	lymph node	23.79	22.70	-	I	III	-	/	/	2021.2 Xinjiang	/	/	S
43	44	45	bronchus	swab	WOAH	31.30	-	-	III	-	-	/	2021.2 Xinjiang	2021.2 Xinjiang	/	S	G
46	47	48	spleen	lymph node	bronchus	-	-	-	-	-	-	2014.4 Zhejiang	2014.4 Zhejiang	2014.4 Zhejiang	S	G	G
49	50	51	WOAH	WOAH	WOAH	32.33	22.54	25.55	I	II	I	/	2024.3 Tibet	/	/	A *	/
52	53	54	swab	spleen	lymph node	25.01	-	18.88	II	-	II	2021.2 Xinjiang	2021.2 Xinjiang	2021.2 Xinjiang	S	G	G
55	56	57	WOAH	lymph node	lymph node	25.69	20.31	-	III	II	-	/	2021.3 Tibet	2021.3 Tibet	/	G	S
58	59	60	lymph node	spleen	lymph node	-	-	-	-	-	-	2020.5 Liaoning	2020.5 Liaoning	2014.6 Shaanxi	S	S	S
61	62		bronchus	bronchus		-	-		-	-		2014.6 Shaanxi	2014.6 Shaanxi		S		

* WOAH: Viral nucleic acid obtained from WOAH. * G: goat; S: sheep; Ga: gazelle; A: antelope.

**Table 6:** Concordance between the newly developed assay and Sanger sequencing for lineage differentiation.

Sample ID			Results of Sanger Sequencing			Sequencing Position			Results of Developed Method (Positive Fluorescence Signal)		
1	2	3	IV	II	IV	Genome	Genome	Genome	ROX	VIC	ROX
4	5	6	III	IV	I	Genome	N gene	Genome	CY5	ROX	FAM
7	8	9	-	IV	IV	-	Genome	F gene	-	ROX	ROX
10	11	12	IV	I	IV	Genome	Genome	F gene	ROX	FAM	ROX
13	14	15	-	-	-	-	-	-	-	-	-
16	17	18	IV	III	IV	Genome	Genome	Genome	ROX	CY5	ROX
19	20	21	IV	-	-	N gene	-	-	ROX	-	-
22	23	24	IV	IV	III	N gene	F gene	F gene	ROX	ROX	CY5
25	26	27	II	-	IV	Genome	-	N gene	VIC	-	ROX
28	29	30	IV	II	IV	Genome	Genome	N gene	ROX	VIC	ROX
31	32	33	IV	IV	-	Genome	F gene	-	ROX	ROX	-
34	35	36	-	IV	-	-	Genome	-	-	ROX	-
37	38	39	IV	-	I	F gene	-	F gene	ROX	-	FAM
40	41	42	I	III	-	Genome	Genome	-	FAM	CY5	-
43	44	45	III	-	-	N gene	-	-	CY5	-	-
46	47	48	-	-	-	-	-	-	-	-	-
49	50	51	I	II	I	N gene	Genome	Genome	FAM	VIC	FAM
52	53	54	II	-	II	Genome	-	Genome	VIC	VIC	VIC
55	56	57	III	II	-	Genome	Genome	-	CY5	VIC	-
58	59	60	-	-	-	-	-	-	-	-	-
61	62		-	-		-	-		-	-	

**Table 7:** Evaluation of diagnostic accuracy.

		Reference Method (R)		
		Positive	Negative	Total
New Method (N)	Positive	38	0	38
	Negative	0	24	24
	Total	38	24	62
Parameter		Value (N/M)	95% CI	
Diagnostic sensitivity		100% (38/38)	90.7–100%	
Diagnostic specificity		100% (24/24)	85.8–100%	
Overall agreement		100% (62/62)	94.2–100%	
Cohen’s kappa		1.00	0.93–1.00	

**Table 8:** Comparison of the developed quadruplex RT-qPCR assay with previously reported PPRV detection methods.

Method	This Study	Xu et al. (2025) [33]	Xu et al. (2025) [15]	Flannery et al. (2019) [26]	Kwiatek et al. (2010) [25]	Tang et al. (2023) [14]	George et al. (2006) [34]
Assay Format	Quadruplex RT-qPCR	Singleplex RT-qPCR	Singleplex RT-qPCR	Singleplex RT-qPCR	Singleplex RT-qPCR	Singleplex RT-qPCR	Multiplex PCR
Target Gene	P	P	H	F	N	L	N and M
Lineage Coverage	Detects and differentiates all lineages	Detects all lineages without differentiation	Detects lineage IV only	Detects all lineages without differentiation	Detects all lineages without differentiation	Detects and differentiates lineage II and IV by analyzing different melting curve analyses.	Detects and differentiates PPRV from RPV (not designed for PPRV lineages)
Primer/Probe System	Single primer pair with 4 probes	Single primer pair with 1 probe	Single primer pair with 1 probe	Single primer pair with 1 probe	Single primer pair with 1 probe	Single primer pair with SYBR Green	Multiple primer pairs
Limit of Detection (LOD)	10 copies/μL	40 copies/μL for lineage I and 4 copies/μL for lineage II-IV	6 copies/μL	10 copies/μL	32 copies	100 copies	10^1^ TCID_50_/mL genes
Multiplex Interference Validation	Systematically evaluated (singleplex vs. multiplex, mixed templates)	Not applicable (singleplex)	Not applicable (singleplex)	Not applicable (singleplex)	Not applicable (singleplex)	Not applicable (singleplex)	Not evaluated in the context of 4 lineages
Key Novelty	First single-reaction assay capable of simultaneous detection and unequivocal differentiation of all four PPRV lineages	Provides a highly efficient method for detecting all lineages but does not differentiate	Provides a highly efficient method for detecting currently predominant epidemic strains	Provides alternative target (F gene) with high in silico sensitivity	Provides alternative target (N gene) with high in silico sensitivity	Differentiate PPRV lineages II from PPRV lineages IV in PPRV infected animals.	Distinguishes PPRV from RPV, not PPRV lineages

## Data Availability

The original contributions presented in this study are included in the article. Further inquiries can be directed to the corresponding author.

## Supplementary Materials

The following supporting information can be downloaded at: https://www.mdpi.com/article/10.3390/ani16091397/s1, Table S1: Details of reference strains used in this study.
